# Evolutionary Footprint: A Systemic Indicator in Evolution, Ecology and Conservation

**DOI:** 10.1111/eva.70267

**Published:** 2026-06-23

**Authors:** Thibault Genissel, Alexandre Robert, Jane Lecomte, François Sarrazin

**Affiliations:** ^1^ Centre d'Écologie et des Sciences de la Conservation, CESCO Sorbonne Université, MNHN, CNRS Paris France

**Keywords:** environmental ethics, evocentrism, evolutionary potential, genetic diversity, global change, macroevolution, microevolution, phylogeny

## Abstract

The growing awareness of evolutionary responses to human‐induced environmental changes highlights the need to better understand and integrate evolutionary perspectives into life sciences and, more broadly, into biodiversity conservation. Evolutionary changes are complex and multi‐causal, operating across numerous timescales. Emerging fields such as eco‐evolutionary dynamics, genomics, and non‐genetic inheritance are substantially advancing knowledge in this area. To better integrate and value these changes, we define the concept of ‘evolutionary footprint’ as the impact of a driver on the micro‐ and macroevolutionary trajectories of a biological entity. By selecting metrics that capture changes in adaptive traits, genetic diversity, macroevolutionary processes, and phylogenetic patterns, and by interpreting the magnitude of these changes relative to natural history baselines, we provide a quantitative method for scoring evolutionary footprints. We illustrate this framework with two case studies, one concerning the evolution of a plant species in response to human‐induced pollinator decline and another addressing potential human impacts on the mammal taxon. We discuss key challenges associated with integrating multiple timescales and levels of biodiversity within the framework, as well as the difficulties of predicting evolutionary changes, accounting for non‐genetic processes, and developing the required databases. This new type of indicator may foster the integration of knowledge about evolutionary processes and eco‐evolutionary dynamics into conservation science and offer new insights and avenues for an evocentric approach to biodiversity conservation.

## 1 Introduction

Over the last three decades, there has been growing awareness among scientists that contemporary evolution plays an important role in driving biodiversity (Western 2001; Carroll et al. 2007; Hendry 2023a). Given that anthropic activities are considered a primary driving force of evolutionary change (Jørgensen et al. 2019; Palumbi 2001), a major challenge to the effective integration of evolutionary perspectives in conservation emerged (Hendry et al. 2010). Recent advances in computational power, high‐speed genomic sequencing data, intelligent data processing, and storage capacity have made it feasible to assess and quantify such evolutionary processes (Winter et al. 2013). This context encourages the integration of evolutionary perspectives—beyond just adaptation (Yamamichi et al. 2023)—into conservation efforts (Hendry et al. 2010; Pelletier and Coltman 2018; Zizka et al. 2022). This paradigm shift in the field of evolutionary sciences (Stockwell et al. 2003; Fronhofer et al. 2023) also provides the opportunity to comprehend the evolutionary impacts of one or several drivers on biological systems, thereby generating novel theoretical insights.

Numerous indicators have been developed to measure what is commonly referred to as the human footprint on biodiversity. In the context of growing awareness of the evolutionary constraints imposed on biodiversity, defining the concept of an Evolutionary Footprint (EvF) is particularly relevant. We use the term *footprint* to denote the specific trace left by a driver on the evolutionary trajectories of other species. This may be applied to any biotic or abiotic driver, although we will mostly consider human activities hereafter. Human activities drive evolutionary responses through eco‐to‐evo effects in an eco‐evolutionary dynamics' perspective (Hendry 2017). Therefore, beyond various scientific interests, understanding the extent of human influence on the evolution of the biosphere is crucial to structure conservation aims and efforts (Sarrazin and Lecomte 2016; IPBES 2022). Indeed, over‐exploitation, land‐use change, invasive species, pollution, and climate change can induce strong microevolutionary responses (Singer et al. 1993; Western 2001; Parmesan 2006; Kendall et al. 2014), and also impact macroevolutionary patterns and processes that play a crucial role in shaping biodiversity (Cowie et al. 2022).

Various frameworks and indicators are currently used in conservation to assess the impact of different drivers on biological entities. For instance, the ecological footprint estimates human impacts on the ecological features of an ecosystem by quantifying the amount of resources that humans can consume without jeopardizing their future consumption (Wackernagel and Rees 1998). However, its accuracy and efficiency have been questioned (Fiala 2008). The biodiversity footprint is a more recent approach that relies on mean species abundance to estimate biodiversity loss (Alkemade et al. 2009). Nevertheless, this indicator has notable limitations, as it fails to capture changes at intraspecific and above‐species taxonomic levels, particularly those related to genetic diversity. Additionally, its interpretation is fully dependent on the definition of an initial species assemblage reference.

These existing footprint indicators could be completed by adopting a more holistic approach of the states and processes governing biological systems. This involves integrating an evolutionary perspective and emphasizing (i) the loss of macroevolutionary diversity, understood as the loss of unique lineages and their accumulated evolutionary divergence (i.e., phylogenetic diversity), (ii) major shifts in evolutionary trajectories, and (iii) the loss of evolutionary potential, rather than simply focusing on the decrease in abundance and the loss of taxonomic diversity or ecological functions. To operationalize this broader evolutionary perspective, it is necessary to consider how evolutionary processes unfold across different scales.

At microevolutionary scales, evolutionary drivers can act through fitness differences (e.g., phenotype‐dependent survival or reproduction), altered gene flow and population structure, mutagenesis, or more complex pathways involving eco‐evolutionary feedbacks (Hendry 2017), epigenetic mechanisms (Ashe et al. 2021), or cultural transmission in social species (Whiten 2019). Although numerous mechanisms may directly or indirectly mediate the effect of a driver on evolutionary change, we do not aim to provide an exhaustive classification here. Instead, we focus on quantifying the evolutionary footprint in terms of changes in trait distributions and genetic diversity.

At macroevolutionary scales, evolutionary processes play a crucial role in shaping biodiversity patterns. Over long timescales, the balance between extinction and speciation events generates the current tree of life and continuously reshapes it (Hedges et al. 2015). Phylogenetic tree topology of taxa captures also key aspects of macroevolution by describing relationships among species within a taxon and identifying groups likely to share traits inherited from common ancestors. Phylogenetic metrics quantify different aspects of this structure, such as the total amount of evolutionary history represented, its distribution among species, and the degree of isolation of particular lineages. Despite uncertainties inherent to phylogenetic construction (Rangel et al. 2015), investigating tree topology is crucial for assessing how drivers affect evolutionary history. We adopt the framework proposed by Tucker et al. (2017), which organizes phylogenetic metrics into three dimensions—richness, divergence, and regularity—to characterize macroevolutionary impacts of drivers.

The term evolutionary footprint has previously been used to describe genomic signatures of long‐term evolutionary processes such as domestication, symbioses, and host‐virus interactions (Pedruzzi et al. 2018; Han and Tsuda 2022). To our knowledge, Pretus (2010) was the first to apply this concept explicitly to human impact on microevolution, although the notion of a significant and systemic human impact on evolution was previously employed in the literature (Kinnison et al. 2007). Other uses of this term with an anthropocentric focus raise concerns about the potential risks to human societies arising from rapid human‐induced evolutionary changes in so‐called “harmful” species (Monosson 2015).

In this paper, we propose a systematic, quantifiable, and accessible framework for scoring the evolutionary footprint (EvF) firmly grounded in ecological and evolutionary sciences. Although it could be used in the same way for a wide range of natural evolutionary drivers, we mostly focus here on the human EvF on biodiversity. The framework is designed to be applicable across various disciplines and relevant to a wide range of conservation stakeholders, combining a semi‐quantitative indicator based on microevolutionary metrics and another based on macroevolutionary metrics.

## 2 Defining the Evolutionary Footprint

We define the evolutionary footprint (EvF) as the effect of a driver, mediated by driving factors, on the microevolutionary and macroevolutionary trajectories of a focal biological entity over a given time scale (Table 1). A driver can be any natural phenomenon, living beings, or group of living beings (e.g., humans), that exert an evolutionary impact on an entity of interest called the focal entity. We use the term driving factor to refer to the specific component of drivers (e.g., hunting, water pollution, increases in mean temperature) that directly change the physical environment; modify ecosystem, community or population structures and processes; or differentially impact individual survival and/or reproduction; thereby resulting in a microevolutionary and/or macroevolutionary response (definition adapted from Pickett and White 1985). Driving factors can be grouped according to standard disturbance ecology criteria including origin, temporal regime, spatial extent and severity (details in Supporting Information S1). The microevolutionary footprint (microEvF) is defined as the divergence in trait distribution and genetic composition of a focal entity between a driver‐exposed scenario andan appropriate driver‐free counterfactual scenario, over a given timescale. It comprises two components: the evolutionary forcing and the changes in evolutionary potential. We define evolutionary forcing as the realized change in the distribution of genetically based traits of a focal entity that is attributable to a driver, quantified through divergence in trait means and/or variances and integrated across major functional trait categories. These changes may arise from directional, diversifying, or stabilizing selection, driver‐induced mutations, founder effects, altered gene flow, amplified genetic drift resulting from driver‐induced population size reduction or other processes. Evolutionary potential can be defined as the property of a biological entity (e.g., genome, trait, population, species) to be able to experience heritable change in some of its components over time (Milot et al. 2020).

**TABLE 1:** Glossary of the terms used in the definition of the evolutionary footprint.

Term	Definition
Driver	Any natural phenomenon, living being, or group of living beings. For instance, *Homo sapiens* species, a beaver population, or an ice age
Driving factors	Productions and activities of a driver implying effective evolutionary response, whether direct or indirect. For instance, human fishing activity, stream flow alteration by beavers, and frost cover during an ice age
Evolutionary trajectory	Entity's phylogenetic, genetic, epigenetic, and cultural variations over time and their associated ecological and phenotypic changes
Micro/macroevolution	Distinction between evolutionary changes typically occurring within a species and the resulting diversity of species on a larger temporal and spatial scale shaped by speciation and extinction. Both processes are powered by the four driving forces of evolution: mutation, genetic drift, gene flow, and natural selection
Focal entity	Any group of living beings of evolutionary interest, whether concrete or abstract. For instance, population, species, or higher taxa
Timescale	Duration chosen for estimating the evolutionary footprint must be within the focal entity's lifespan and in such a way that the driver's potential impact is included in this duration

The macroevolutionary footprint (macroEvF) is defined as the divergence in species composition and phylogenetic diversity of a focal species assemblage between a driver‐exposed and an appropriate driver‐free counterfactual scenario, over a given timescale. This divergence is quantified through changes in extinction and speciation rates and the associated changes in phylogenetic tree topology.

Establishing the effect of a driver on evolutionary trajectories inherently requires comparing evolutionary metrics between a scenario with the driver and a reference scenario without it. This reference scenario can be observed, reconstructed, or model‐based and serves as the baseline (Robert 2024). Given the properties of ecosystem ecology and evolution, such references should not be viewed as static states but rather as dynamic processes (Hiers et al. 2012). Explicitly defining reference baselines is particularly important in conservation science, as poorly specified baselines can strongly bias inference (White and Walker 1997; Balaguer et al. 2014; Robert 2024).

For the microEvF, the reference may be (i) a similar entity (e.g., synchronic design using ecologically similar population) that has not experienced the focal driver, (ii) the same focal entity prior to the driver's impact (e.g., an allochronic design using the same population at different times, including resurrection approaches; see Box 1) (Hendry and Kinnison 1999), or (iii) a model‐based counterfactual scenario when no suitable reference is available. When such temporal data are not available, a synchronic study must be conducted using another population of common origin that has not been exposed to the driver's pressure. Constructing counterfactual scenarios therefore requires prediction, which represents a particularly challenging epistemological task in science, and especially in evolutionary biology (Hendry 2023b; Mouquet et al. 2015). Evolutionary predictability and repeatability are generally low and are influenced by many factors (Wortel et al. 2023). While theoretical approaches based on quantitative genetics are available, accurate predictions still rely on experimental or long‐term empirical studies (Shaw 2019).

BOX 1 **Microevolutionary footprint on a plant population: Case study of *Viola arvensis* Murray.**

The evolutionary response of flowering plants to the absence of pollinators has been modelled (Porcher and Lande 2005) and then evidenced in a controlled environment (Bodbyl Roels and Kelly 2011). More recently, this evolutionary response has been documented under natural conditions in the field pansy ( *Viola arvensis* ), in correlation with the decline in pollinator activity (Acoca‐Pidolle et al. 2024). The field pansy is an herbaceous annual flowering plant that reproduces by seed either through pollination or selfing (self‐pollination). The study used a resurrection ecology approach, comparing ancestral and descendant populations grown under common conditions using seeds stored since the 1990s. The evolutionary changes occurred over at most 30 generations, corresponding to 30 years. Acoca‐Pidolle et al. (2024) reported an increase in selfing rate, a reduction in floral display (including corolla size and related floral traits), and a decrease in nectar volume, all of which were associated with reduced attractiveness to bumblebees. In contrast, vegetative traits showed no detectable change, and no significant reduction in overall genetic diversity was observed (Table 3, Figure 2). These evolutionary changes in the *Viola arvensis* population were attributed to reduced pollinator activity caused by anthropogenic activities and human‐induced changes such as habitat loss and fragmentation, climate change, agrochemical use, and invasive species (Potts et al. 2010; Thomann et al. 2013).

To compute the microevolutionary footprint, changes in the mean and variance of trait distributions were expressed as standardized effect sizes (Hedges's *d*) and variability ratio between ancestral (reference) and descendant (driver) populations, and genetic diversity was assessed using a single population‐level diversity metric, expressed as a log‐ratio between driver and reference scenarios. The metrics used are presented in Table 2 and were calculated based on the published data.

**TABLE 2:** Metrics, formula and classification of metric values for the evolutionary footprint framework for the micro and macroevolutionary scales.

Scale	Category	Metric	Formula	Classification of metric values				
				Negligible	Small	Medium	Large	Very large
Microevolutionary footprint	Trait distribution	Change in mean (Hedge's *d*)	$\text{ΔMean}=\left|\frac{{\bar{X}}_{\text{driver}-}{\bar{X}}_{\mathrm{ref}}}{{\mathrm{SD}}_{\text{pooled}}}\;\right|J$	0–0.1	0.1–0.5	0.5–0.8	0.8–1.3	> 1.3
			${\mathrm{SD}}_{\text{pooled}}=\sqrt{\frac{\left(n_{\mathrm{ref}}-1\right){\mathrm{SD}}_{\mathrm{ref}}^{2}+\left(n_{\text{driver}}-1\right){\mathrm{SD}}_{\text{driver}}^{2}}{n_{\mathrm{ref}}+n_{\text{driver}}-2}}$ $\;J=1-\frac{3}{4\left(n_{\text{driver}}+n_{\mathrm{ref}}-2\right)-1}$					
		Change in variance (variability ratio)	$\text{ΔVar}=\left|\ln\;\left(\frac{{\mathrm{SD}}_{\text{driver}}}{{\mathrm{SD}}_{\mathrm{ref}}}\right)+\frac{1}{2\left(n_{\text{driver}}-1\right)}-\frac{1}{2\left(n_{\mathrm{ref}}-1\right)}\right|$	0–0.1	0.1–0.2	0.2–0.5	0.5–0.8	> 0.8
	Genetic diversity	Change in genetic diversity (log ratio) (e.g., heterozygosity, allelic richness…)	$\mathrm{\Delta GD}=\left|\ln\left(\frac{{\mathrm{GD}}_{\text{driver}}}{{\mathrm{GD}}_{\mathrm{ref}}}\right)\right|$	0–0.05 (0%–2%)	0.05–0.10 (2%–10%)	0.10–0.30 (10%–25%)	0.30–1.4 (25%–75%)	> 1.4 (> 75%)
Macroevolutionary footprint	Diversification dynamics	Change in extinction rate (log ratio)	$R_{\mu }=\left|\ln\left(\frac{\mu_{\text{driver}}}{\mu_{\mathrm{ref}}}\right)\right|$	0–0.7 (0–2×)	0.7–1.6 (2–5×)	1.6–2.3 (5–10×)	2.3–4.6 (10–100×)	> 4.6 (> 100×)
		Change in speciation rate (log ratio)	$R_{\lambda }=\left|\ln\left(\frac{\lambda_{\text{driver}}}{\lambda_{\mathrm{ref}}}\right)\right|$	0–0.7 (0–2×)	0.7–1.6 (2–5×)	1.6–2.3 (5–10×)	2.3–4.6 (10–100×)	> 4.6 (> 100×)
	Phylogenetic structure	Change in phylogenetic diversity metric (ratio) (e.g., Faith's PD, ED, MPD…)	$\mathrm{\Delta PM}=\left|\frac{\left({\mathrm{PM}}_{\text{driver}}-{\mathrm{PM}}_{\mathrm{ref}}\right)}{{\mathrm{PM}}_{\mathrm{ref}}}\right|$	0%–2%	2%–8%	8%–20%	20%–40%	> 40%

*Note:* At microevolutionary scale, trait distribution and genetic diversity changes are presented. Hedges' *d* formula is used to measure change in mean for a given trait (Nakagawa et al. 2015), here noted ∆Mean. Variability ratio (cf. Hedges and Nowell 1995), here noted ∆Var, is used to measure change in variance for a given trait distribution. ${\bar{X}}_{\text{driver}}$ and ${\bar{X}}_{\mathrm{ref}}$ are the trait means in the focal entity under the driver's pressure and in the reference scenario, respectively. SD_driver_ and SD_ref_ are the standard deviations of the trait distributions, *n*_driver_ and *n*_ref_ are the corresponding group sizes. J is a bias correction for small sample sizes. Without this correction, Hedges' *d* becomes Cohen's *d*. The absolute log‐ratio value is used to measure change in genetic diversity, noted ΔGD, between the genetic diversity of the entity under the driver's pressure (GD_driver_) and in the reference scenario (GD_ref_). At the macroevolutionary scale, change in extinction rate, noted $R_{\mu }$, is measured with the absolute value of the log‐ratio between the extinction rate within the focal entity under the driver's pressure, noted $\mu_{\text{driver}}$, and the extinction rate in the reference scenario, noted $\mu_{\mathrm{ref}}$. Change in speciation rate, noted $R_{\lambda }$, is measured with the same method. Finally, phylogenetic structure change, noted 𝛥PM, is measured as the relative change in percentage between the phylogenetic diversity metric value of the entity under the driver’s pressure, noted PM_driver_, and the phylogenetic diversity metric value in the reference scenario, noted PM_ref_. The phylogenetic diversity metrics (e.g., Faith’s PD, ED, MPD...) are categorized as in Tucker et al. (2017). After computation, values are classified into 5 categories from negligible to very large. Categories for change in mean (effect size) are based upon categories from Sullivan and Feinn (2012). The other categories have been adapted to the corresponding metrics.

The metric values for each group of traits have been assigned a magnitude category according to the classification scale presented in Table 2. Floral traits (e.g., floral area approximation and nectar volume), mating system traits (selfing rate), and phenological traits (anthesis duration) fall in small or medium categories, whereas changes in genetic diversity fall within the negligible category (Figure 2). Accordingly, the evolutionary forcing score (F) is medium, the evolutionary potential change score (P) is negligible, and the timescale category is T_2_, for a number of generations between 5 and 20. The resulting microEvF profile is therefore {F_Medium_, P_Negligible_, T_2_}. The microEvF score is the average between F and P scores, plus an optional timescale adjustment. Here, F score is 2 (medium), P score is 0 (negligible), and timescale category T_2_ increases the score by 0.5. So, the microEvF score is 1.5.

In this case, the driver is characterized by substantial evolutionary forcing over a short timescale, while the reduction in evolutionary potential remains limited at present. However, the observed increase in selfing rate is interpreted by the authors as a potential precursor of future genetic erosion, illustrating an evolutionary footprint debt. As suggested by the authors, a positive eco‐evolutionary feedback loop may be occurring: the reduction in pollinator activity leads to a decrease in flower size and nectar production, which in turn reduces plant attractiveness to pollinators and potentially jeopardizes pollinator survival. Such a downward spiral could lead to severe evolutionary disruption of a fundamental mutualistic interaction in the ecosystem, thus potentially resulting in the rapid extinction of the plant or pollinator populations or the loss of genetic diversity within the plant population, ultimately leading to extinction in the long term (Cheptou 2019).

This case study highlights both the strengths and limitations of the EvF framework. Flowering plants frequently evolve toward a selfing syndrome, a shift that carries complex evolutionary consequences, mostly adaptive in the short term but potentially detrimental over the long term (Takebayashi and Morrell 2001; Tsuchimatsu and Fujii 2022). Since the rate of evolutionary change can differ between traits and between species (Carleial et al. 2017), standardized and comparable metrics are required to evaluate whether observed changes are exceptional relative to background evolutionary dynamics (Hansen et al. 2024). The *V. arvensis* example illustrates how the EvF framework can integrate trait evolution, genetic diversity, and timing to provide a synthetic assessment of contemporary evolutionary change, while explicitly acknowledging remaining uncertainties.

For the macroEvF, the reference consists of background evolutionary processes—that is, extinction and speciation rates in the absence of the focal driver—and the corresponding pre‐driver values of phylogenetic metrics. Paleontological data provide valuable references on the background extinction and speciation rates and taxa diversification (Alroy 2014; De Vos et al. 2015). When paleontological data are unavailable or incomplete, estimation models can be used (Paradis 2004; Silvestro et al. 2014), or, with caution, reference values may be approximated from closely related taxa.

Evolutionary footprint may arise from direct effects of a driver on the focal entity, or from indirect effects mediated by other species (e.g., invasive species or altered predators and mutualists). In both cases, the resulting footprint is attributed to the original driver.

The temporal dimension of the evolutionary footprint is critical and must be defined carefully. We first distinguish *realized EvF*, which corresponds to the evolutionary divergence that has already occurred between the trajectory shaped by the driver and the reference scenario without it over a given timescale.

In addition, the EvF includes a future component that we decompose into two parts

- We define an *EvF debt* as future evolutionary divergence that is already locked in by past and current pressures, even if the driver were to cease now. For example, at the microevolutionary scale, a substantial loss of genetic diversity in a population represents both a realized footprint and an EvF debt, as it constrains future adaptive potential and increases the probability of subsequent trait change or extinction.
- We also define *a scenario‐based future EvF*, which corresponds to the additional footprint expected if the driver continues to operate according to a specific scenario (e.g., business‐as‐usual land‐use change, climate projections). At the macroevolutionary scale, forecasts of mammal extinctions and loss of phylogenetic diversity under continued human pressures by 2100 provide a clear example of such scenario‐based future EvF (see Box 2).

BOX 2 **Macroevolutionary footprint: Case study of mammal taxa.**

The impact of human activities on mammal abundance and diversity is well documented (Faurby and Svenning 2015; Andermann et al. 2020), providing clear evidence of a substantial macroevolutionary footprint of human‐induced pressures on the mammal taxon. Available projections further suggest that this footprint will deepen over the coming decades and centuries as threats to mammal diversity intensify (Jono and Pavoine 2012). To illustrate these projected macroevolutionary changes, we assessed the macroevolutionary footprint score of the human driver on mammal taxa under a projected scenario for the year 2100 (Figure 3, Table 3), using a pre‐industrial reference state taken in 1500 ce. This reference corresponds to 600 years of cumulative driver impact. The metrics are based on current and limited knowledge and therefore likely underestimate both the total number of threatened species and the magnitude of future loss.

**TABLE 3:** Detailed data presented in Figures 2 and 3 for the computation of the micro‐ and macroevolutionary footprint scores. Values represent the magnitude of change between the entity under driver pressure and the reference scenario. Magnitude of change categories range from negligible to very large, following the classification defined in Table 2. In the “Magnitude of change” column, values in italics refer to the magnitude of individual sub‐metrics (|Δmean| and |Δvar|), while values in regular font refer to the overall magnitude assigned to the trait group, corresponding to the highest magnitude level among its sub‐metrics. NA indicates that the metric was not applicable or not assessed for the corresponding group. “No data” indicates that data were not available for the given metric.

Scale	Category	Group	Metric	Value	Magnitude of change
Microevolutionary footprint (Figure 2)	Traits^a^	Morphology	Floral area		Medium
			|Δmean|	0.52	*Medium*
			|Δvar|	0.24	*Medium*
		Physiology	Nectar volume		Medium
			|Δmean|	0.35	*Small*
			|Δvar|	0.24	*Medium*
		Phenology	Anthesis duration		Small
			|Δmean|	0.38	*Small*
			|Δvar|	0.15	*Small*
		Life History Trait	Selfing rate		Small
			|Δmean|	0.28	*Small*
			|Δvar|	NA	
		Behaviour	NA		
	Genetic diversity^a^	Genetic diversity	Expected heterozygosity	Not significant	Negligible
Macroevolutionary footprint (Figure 3)	Diversification dynamics^b^		Extinction rate^2^	×30,260	Very large
			Number of species^2^	15%	NA
			Speciation rate	No data	
	Phylogenetic diversity^c^	Phylodiversity richness	Faith's PD	13%	Medium
		Phylodiversity divergence		No data	
		Phylodiversity originality		No data	

^a^ Data from Acoca‐Pidolle et al. (2024).

^b^ Data from Andermann et al. (2020).

^c^ Data from Gumbs et al. (2020).

Based on simulations of IUCN Red List trends, Andermann et al. (2020) estimated future changes in mammalian diversity and extinction rates for 2100 ce relative to a deep‐time baseline (126 ka ago). This baseline yields slightly higher estimates of evolutionary change than those obtained using a more recent pre‐industrial reference, but it robustly highlights the dominant role of humans as an evolutionary driver, including during early phases of human influence. According to their results, the number of mammal species decreased from 6065 species 126 ka ago to 5156 in 2100 ce, representing a reduction of ~15%. Extinction rates increased from 3.894 × 10^−8^ 126 ka ago to 1.178 × 10^−3^ extinctions per species per year in 2100 ce, corresponding to an increase of more than four orders of magnitude (30,260‐fold).

Projected losses of phylogenetic diversity (PD) further illustrate the depth of this macroevolutionary footprint. Estimates indicate that approximately 6.4 billion years of mammalian phylogenetic diversity could be lost by 2100, corresponding to a reduction of ~13% in PD richness (Gumbs et al. 2020). Comparable projections suggest losses of ~7% for birds and ~ 16% for amphibians.

Human activities may also influence speciation processes (Bull and Maron 2016), although quantitative estimates of human‐induced speciation rates remain scarce and highly uncertain. Background speciation rate estimates range between 0.05 and 0.2 new species per species per million years (De Vos et al. 2015), with a commonly cited value of 0.07 for mammals (Pimm et al. 2014). Human‐induced speciation is difficult to quantify because many processes are ongoing and species delimitation remains challenging. Domestication represents one clear pathway of human‐driven diversification, with at least 17 mammal species domesticated by humans (Burgin et al. 2018). This process has generated numerous domesticated lineages and may ultimately lead to speciation events, such as the potential future divergence of domestic dogs ( *Canis lupus familiaris* ) from grey wolves ( *Canis lupus lupus* ). If domesticated and wild subspecies were to diverge rapidly into distinct species, human‐induced speciation rates over the past 20,000 years could exceed background levels. Additionally, human‐altered niches, including urban environments (Szulkin et al. 2020), can promote divergence and speciation, as illustrated by the urban speciation of the London Underground mosquito, *Culex pipiens f. molestus* (Thompson et al. 2018).

To construct the macroevolutionary EvF indicator, we first evaluated the magnitude of human‐induced macroevolutionary change using two complementary components. Changes in diversification dynamics were assessed through changes in extinction and speciation rates. Projected mammalian extinction rates by 2100 indicate a very large increase relative to background levels, whereas changes in speciation rates remain comparatively limited and uncertain. Changes in macroevolutionary potential were evaluated through projected losses in phylogenetic diversity, which reflect a substantial erosion of evolutionary history and future diversification potential. According to the categories defined in the EvF framework, the 16% reduction in phylogenetic diversity corresponds to an intermediate level of change. Accordingly, the macroevolutionary footprint profile for mammals is characterized by a very large change in diversification dynamics (D), medium change in phylogenetic structure (S), and the timescale category T_3_, for long‐term projection. The macroEvF profile is then {D_Very large_, S_Medium_, T_3_}. The macroEvF score is the average of the D and S score plus a T score adjustment. Here, D score is 4 (very large), S score is 2 (medium), so, the resulting macroEvF score is 3 (Figure 3).

## 3 Scoring the Microevolutionary Footprint

**FIGURE 1:**
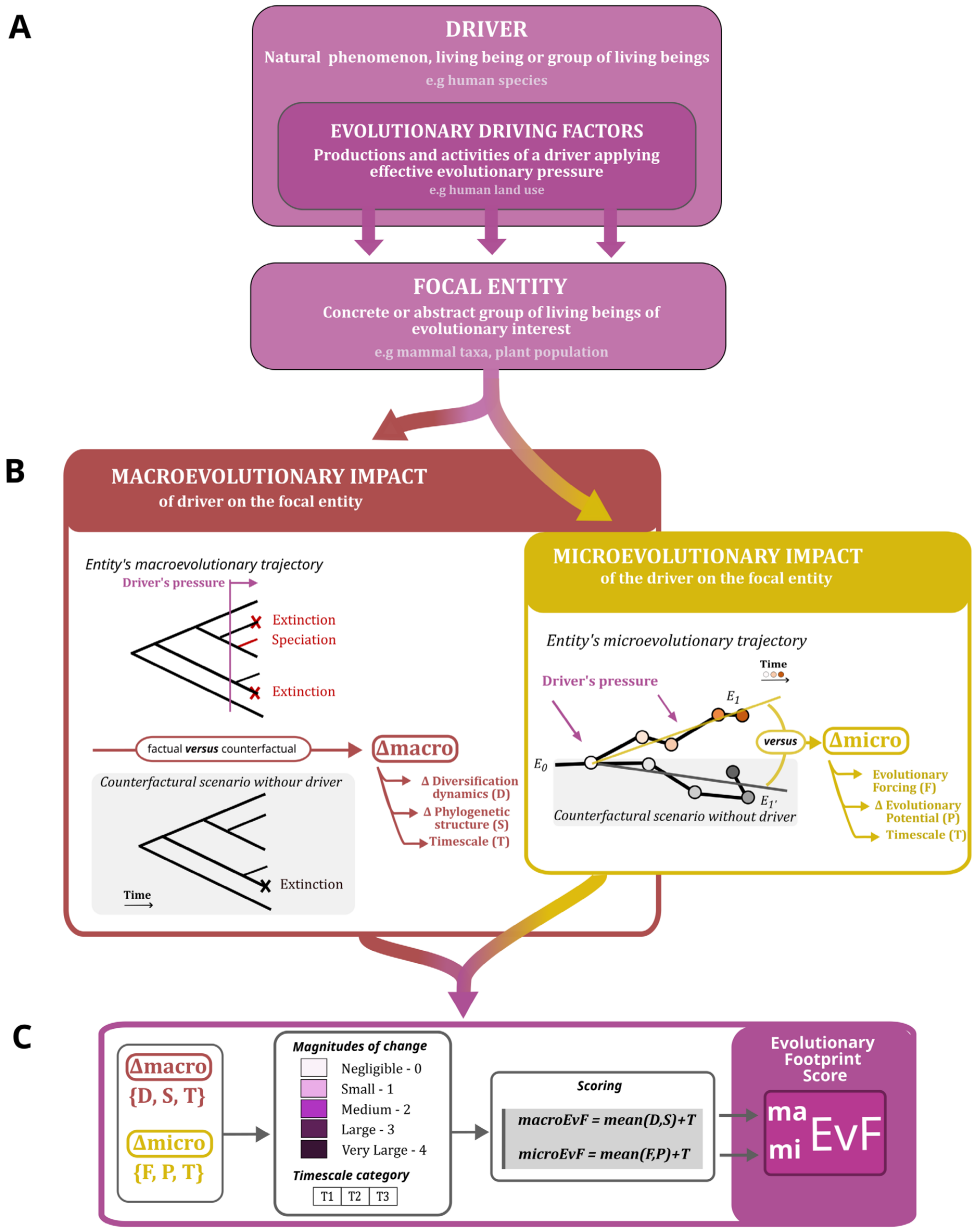
Main steps for scoring the evolutionary footprint of a driver on a focal entity. (A) Definition of the driver and focal entity on which evolutionary pressure is exerted. (B) Schematic representation of the macroevolutionary and microevolutionary impacts of the driver on the focal entity. The macroevolutionary impact (Δmacro) refers to changes, over a given timescale (T), in diversification dynamics (D)—including extinction rate, speciation rate, species richness—and in phylogenetic structure (S), including phylogenetic richness, divergence, and regularity, relative to a counterfactual scenario without driver pressure. The microevolutionary impact (Δmicro) refers to the effect of the driver on the entity's microevolutionary trajectory, represented here with phenotypic evolution vectors (E_1_) (as described by Adams and Collyer (2009)), during a given timescale (T), relative to a reference scenario, through evolutionary forcing (F), measured with the change in trait distribution and the change in evolutionary potential (P), correlated with the change in genetic diversity. (C) The different metric values obtained are assigned to categories of magnitude of change, ranging from negligible (0) to very large (4). For each micro‐ and macroevolutionary category—evolutionary forcing, evolutionary potential, divergence dynamics and phylogenetic structure—the highest magnitude level among internal metrics is retained. The microevolutionary footprint score is calculated as the mean of the scores of evolutionary forcing and evolutionary potential change, plus an additional value depending on the timescale considered. Similarly, the macroevolutionary footprint score is calculated as the mean of the scores of diversification dynamics and phylogenetic structure changes, plus a timescale‐dependent adjustment. The combined micro‐ and macroevolutionary scoring yields the overall evolutionary footprint score, where ma denotes the macroevolutionary footprint score and mi the microevolutionary footprint score.

We aim to provide a score for the driver's EvF depth to serve as an integrative indicator. The IUCN Red List of Endangered Species relies on a system of categories and criteria, which considers multimodal thresholds using a set of demographic metrics (population size, geographic range, small populations dynamics, etc.), depending on the availability of data, to assess extinction risk (IUCN 2023). The EvF framework adopts a similar integrative and flexible approach to scoring both microevolutionary and macroevolutionary footprints (Figure 1).

**FIGURE 2:**
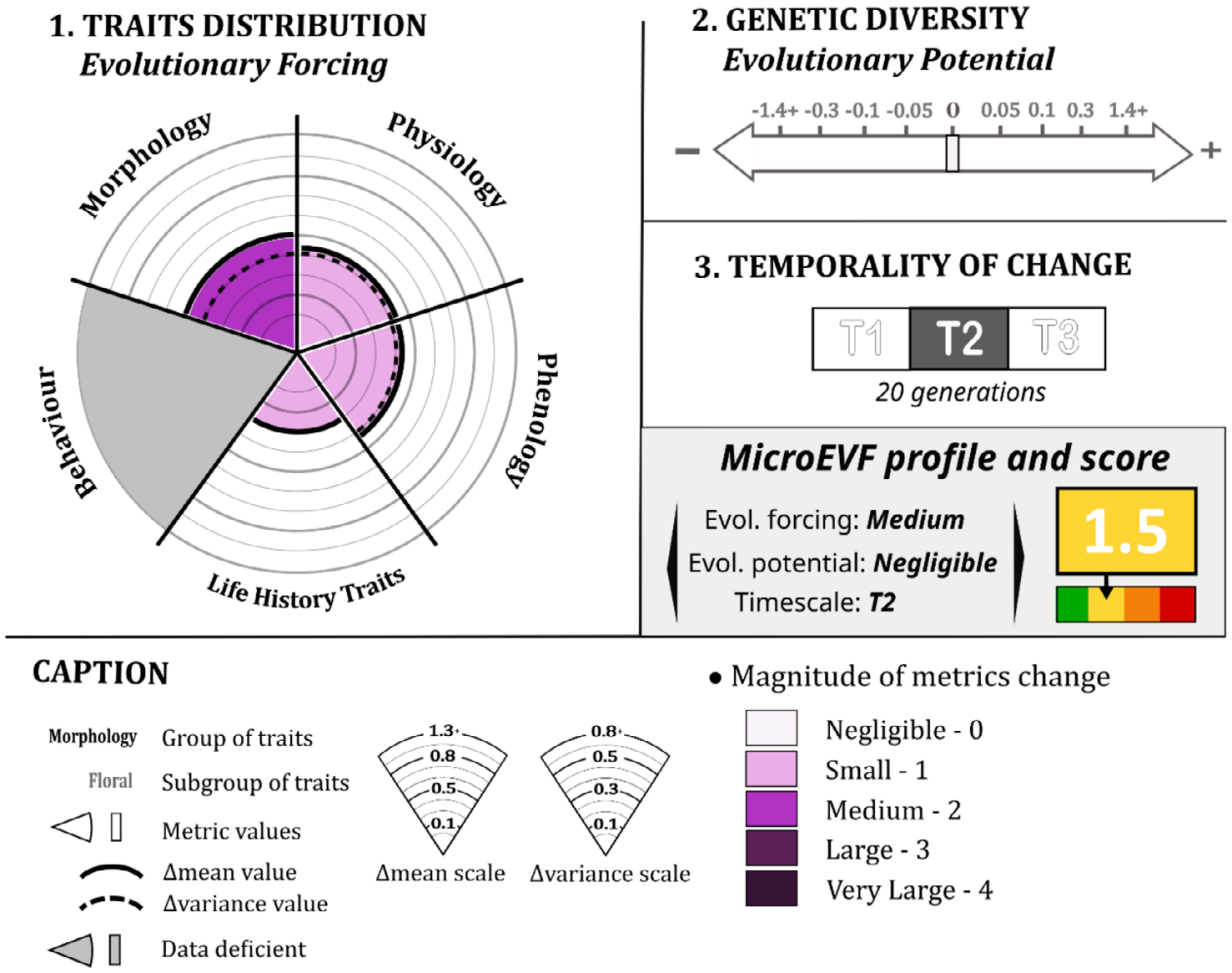
Multidimensional indicator for the microevolutionary footprint, illustrating the case of the indirect human footprint on a plant species, *Viola arvensis*, through pollinator decline over 30 years, assessed using a resurrection protocol (Acoca‐Pidolle et al. 2024). Representation of the three components of the microevolutionary footprint: changes in trait distributions (1), changes in genetic diversity (2), and the timescale of change (3). Traits are classified into five broader categories: physiology, life history traits, morphology, phenology, and behavior. Metric values are averaged within categories. For each group, the standardized mean difference (∆mean) between the driver and reference scenarios (plain line), and the effect size for variance based on the log‐ratio of standard deviations (∆var), with a size correction, are reported (dotted line). Metrics considered include the floral area (morphology), nectar production (physiology), selfing rate (life history trait) and anthesis duration (phenology). Changes in genetic diversity change is represented as a unidimensional metric, calculated as the logarithmic ratio of a genetic diversity measure between the driver and reference scenarios, here, expected heterozygosity (He) is used. Detailed data are provided in Table 3. The timescale of change is categorized into three categories (T1: *t* ≤ 5 generations, T2: 5 < *t* ≤ 20, T3: *t* > 20). Metric values are assigned to categories of magnitude of change from negligible to very large. Evolutionary Forcing and Evolutionary Potential are assigned the highest magnitude level observed among their internal metrics (trait distribution change for the evolutionary forcing and genetic diversity change for the evolutionary potential). Here, the magnitude for the evolutionary forcing is *medium* and the magnitude for the evolutionary potential change is *negligible*. The microevolutionary footprint profile is expressed as the triplet {F,P,T} with the associated magnitude level for each component. Here, the triplet is {F_Medium_, P_Negligible_, T_2_}. Finally, the overall microevolutionary footprint score is calculated as the mean of the magnitude level of F and P, plus an additional value depending on the timescale T considered. In this case, F score is 2 (*medium*), P score is 0 (*negligible*), T2 increases the score by 0.5, resulting in a microEvF score of 1.5.

Evaluating the magnitude of a driver's microevolutionary footprint implies focusing on three complementary levels: (i) changes in the distributions of traits that will define the evolutionary forcing magnitude (F), (ii) changes in genetic diversity in the focal population, which define the magnitude of change in evolutionary potential (P), (iii) and the timescale (T) over which these changes have occurred, expressed in number of generations or years (Figure 2).

### 3.1 Measuring the Changes in Trait Distribution and Determining the Evolutionary Forcing

Trait‐based metrics quantify the extent to which the distribution of a focal trait (or set of traits) diverges between a scenario with the driver and the reference without it. A well‐documented human‐induced evolutionary change in a wild population is the phenotype‐based selective harvest of bighorn sheep in Canada (Coltman et al. 2003). In this system, breeding values for body weight and horn length were monitored over 30 years in a hunted population, revealing selection against males with rapidly growing horns and rapid phenotypic change. To measure the changes in trait distributions, and for the sake of simplicity, we propose to quantify for each trait, (i) a standardized mean difference, using Hedges' *d* value (Nakagawa et al. 2015), between the entity under the driver's pressure and in the reference scenario, and (ii) a variability ratio (Hedges and Nowell 1995) to measure the change in variance (see formula in Table 2).

To use these effect sizes within a semi‐quantitative indicator, we do not rely on their raw values but classify them into ordinal magnitude classes. For each trait, we first convert the absolute standardized mean difference ∣Δmean∣ into five categories, following commonly used thresholds in statistics (Ferguson 2009; Sullivan and Feinn 2012): negligible (0–0.1), small (0.1–0.5), medium (0.5–0.8), large (0.8–1.3) and very large (> 1.3). In order to extensively capture the change in trait distribution, we evaluate the change in variance of the trait. We apply an analogous ordinal classification to the absolute variance effect ∣Δvar∣: negligible (0–0.1), small (0.1–0.2), medium (0.2–0.5), large (0.5–0.8); very large (> 0.8). Although no formal convention exists for classifying variance changes, these thresholds are pragmatic and can be refined in future applications. This procedure yields two categorical scores per trait (Table 2). The magnitude level of change in trait distribution is then defined as the highest category obtained from the mean and variance effects, ensuring that substantial variance changes are not overlooked when mean values remain stable, for example under disruptive selection. An illustrative case is the Windermere pike ( *Esox lucius* ), in which harvest‐induced disruptive selection led to an increase in phenotypic variance in growth‐related traits (Edeline et al. 2009).

Because the driver's pressure tends to act on a group of traits with correlated functions, we suggest considering traits within five broader groups: *morphology*, *life history traits*, *phenology*, *behavior*, and *physiology*. At a minimum, data for one trait per category should be included. Within each group, standardized mean effect and the log‐ratio of standard deviations should be computed for each trait, averaged, and then classified as described above. This results in five categorical scores describing changes in trait distribution across major functional dimensions. Within each functional trait group (e.g., morphology, behaviour, etc.), the framework allows the definition of evolutionarily or functionally coherent sub‐groups in order to reduce trait dilution effects. Changes are aggregated within each sub‐group, and the magnitude of change retained for the functional group is then defined by the maximum level observed among its sub‐groups, ensuring that strong signals from key traits are preserved.

Within the EvF scoring framework, the *evolutionary forcing* is captured through its combined effect on trait distributions rather than through the underlying mechanisms themselves. The magnitude of evolutionary forcing (F) is defined as the highest classification of change among the five trait groups (negligible, small, medium, large, or very large).

### 3.2 Measuring the Changes in Genetic Diversity and Determining the Change in Evolutionary Potential

Drivers can also affect microevolution by altering genetic diversity. Intraspecific genetic diversity reflects the magnitude of genetic variability within a population (Hughes et al. 2008). It underpins evolutionary and ecological processes (Mimura et al. 2017), contributes to fitness and adaptive potential in changing environments (Hoffmann et al. 2017), and can influence ecosystem productivity and species interactions at the community level (Crutsinger 2016).

To operationalize changes in genetic diversity within the EvF framework, we express the impact of a driver as the logarithmic ratio of the selected genetic diversity metric between the driver and reference scenarios (Table 2). This formulation allows symmetrical treatment of increases and decreases in genetic diversity on a common scale, acknowledging that both gains and losses constitute evolutionary footprints with equal weight assigned to both increases and decreases in genetic diversity, as captured by the log‐ratio change. Indeed, although reductions in genetic diversity are typically associated with increased extinction risk and reduced evolutionary potential, increases in diversity may also reflect strong driver‐induced processes such as admixture, hybridization, and therefore equally represent a footprint on evolutionary trajectories. The specific genetic diversity metric (e.g., heterozygosity, nucleotide diversity, allelic richness) can be chosen according to data availability, marker type, and study objectives, provided it is applied consistently in the driver and reference scenarios. The resulting log‐ratio is classified into ordered magnitude categories that reflect increasing deviation from the reference state: negligible (< 0.05), small (0.05–0.1), medium (0.1–0.3) and large (0.3–1.4) and very large (≥ 1.4) (Table 2). These categories serve as comparative classes within the EvF framework rather than absolute threshold. The assigned class defines the genetic diversity component of the evolutionary potential score (P) within the (F, T, P) triplet, in direct parallel with the trait‐based evolutionary forcing component. We assume that the magnitude of change in evolutionary potential (P) corresponds to the classification of genetic diversity change (negligible, small, medium, large or very large).

### 3.3 Determining the Timescale of Microevolutionary Changes

We deliberately treat time as a separate dimension rather than embedding it directly into trait‐level effect sizes, and instead use it to modulate the interpretation of forcing and potential at the scoring stage.

In addition to the magnitude of changes in trait distributions and genetic diversity, the timescale over which divergence occurs is critical for interpreting the magnitude of the evolutionary footprint. For instance, a shift of one standard deviation in a focal trait over three generations does not carry the same meaning as the same shift over thirty generations: for a given magnitude of change, shorter timescales imply stronger forcing. In principle, this can be formalized through evolutionary rates expressed in various metrics (Gingerich 1983; Roopnarine 2003; Hunt 2012) such as Haldanes, which quantify evolutionary rate as the change in the mean of a trait in units of within‐population standard deviations per generation (Haldane 1949). However, because the EvF framework is intended as a comparative indicator across very different taxa and study designs, we treat time as a separate dimension rather than embedding it directly into trait‐level effect sizes.

We classify the timescale of observed changes into four broad categories (T0: unknown, T1: short, T2: medium, T3: long), based on the approximate number of generations over which the driver has acted (T1: < 5 generations, T2: 5–20, T3: > 20). Short timescales (T1) capture rapid changes likely associated with strong selective pressures, which are of particular concern for conservation due to limited time for adaptive responses. Intermediate timescales (T2) correspond to commonly documented situations in terms of data availability, where evolutionary pressure can still lead to significant biological outcomes. Longer timescales (T3), more easily assessed in taxa with short generation times but less frequently documented in other taxa, can highlight deeper evolutionary changes with lasting effects on genetic diversity and adaptive potential. These categories are designed to balance biological relevance with operational simplicity, and to support comparison across case studies while remaining adaptable to ecological context and data availability. When generation time is unknown, approximate years may be used as a proxy and should be reported explicitly.

### 3.4 Scoring the Microevolutionary Footprint

To summarize, each driver–entity combination is characterized by an EvF scoring profile (F, P, T), composed of ordinal magnitude classes describing evolutionary forcing on the focal entity (F), change in evolutionary potential of the focal entity (P), and the timescale (T) category over which F and P occurred. We also propose an integrative scoring procedure that summarizes this triplet into a single numerical score ranging from 0 to 4. To do so, we propose a simple and transparent aggregation rule. First, the forcing (F) and potential (P) scores—each ranked from 0 (negligible) to 4 (very strong)—are averaged to reflect the overall magnitude of the microevolutionary response. This averaged value is then adjusted according to the temporal category of the driver's impact. Because rapid evolutionary changes are of particular concern in conservation, a timescale‐dependent weighting is applied to the score when the score is above 0: +1 for very rapid change (T1), +0.5 for intermediate timescales (T2), and no adjustment for slow change (T3).

This three‐dimensional description facilitates interpretation of contrasting situations. High forcing combined with a strong decline in evolutionary potential over short timescales represents a critical case in which rapid change and reduced adaptive capacity are symptomatic of a deep EvF. By contrast, high forcing acting on short timescales in populations that retain high evolutionary potential may indicate a window of opportunity for intervention. Finally, low forcing acting over long timescales in a system with already low evolutionary potential is more consistent with historical legacies of past pressures than with strong contemporary impacts.

We illustrate the microevolutionary footprint scoring using a plant case study (Box 1; Figure 2). We used data obtained by Acoca‐Pidolle et al. (2024) to estimate the depth of the microevolutionary footprint associated with decline in pollinator abundance in a population of field pansy. Applying the EvF scoring method yielded the microEvF profile: (F_Medium_, P_Negligible_, T_2_), corresponding to an overall score of 1.5. This case illustrates strong and relatively rapid evolutionary forcing that has not yet measurably affected evolutionary potential.

## 4 Scoring the Macroevolutionary Footprint

**FIGURE 3:**
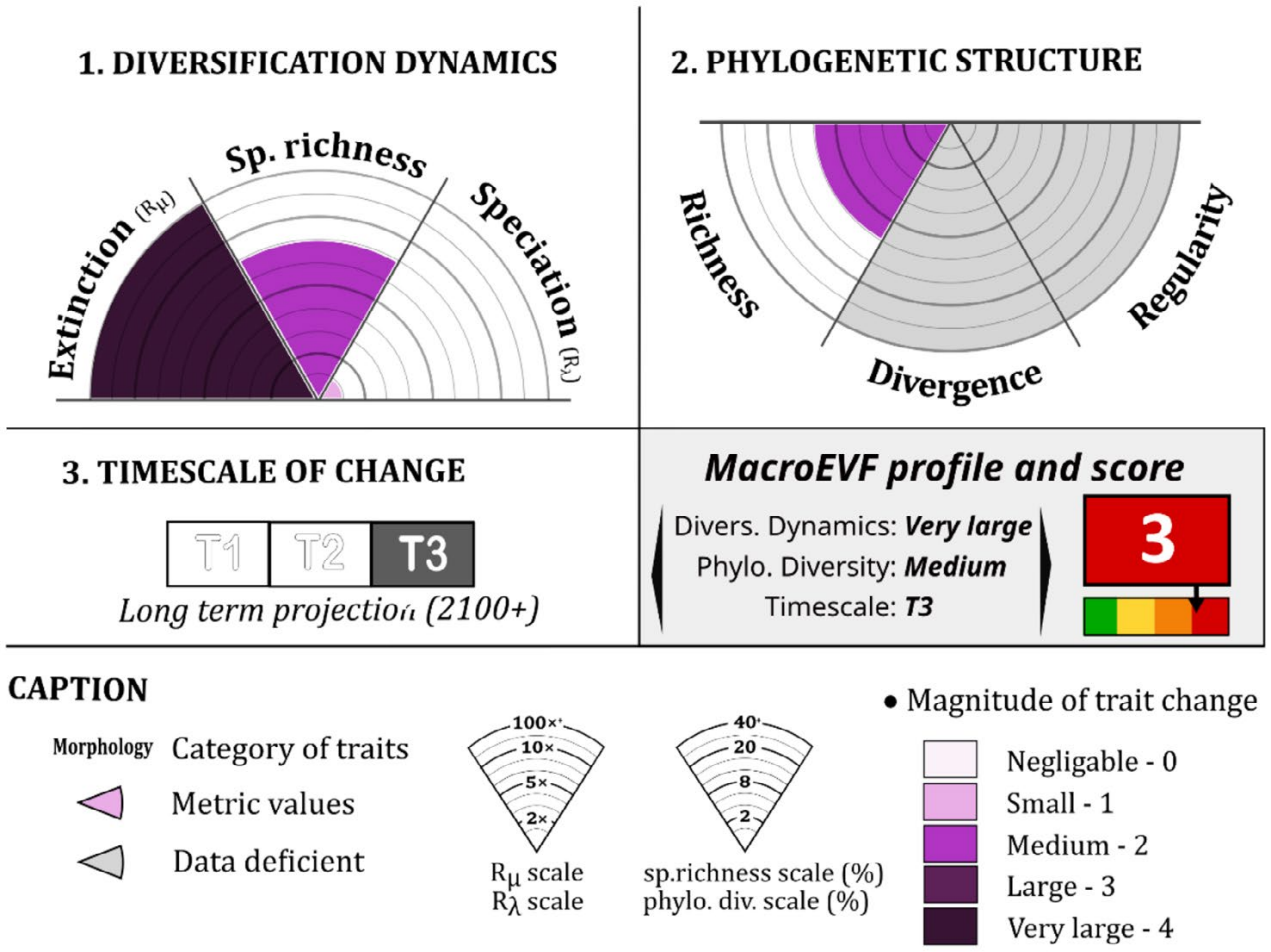
Multidimensional indicator for the macroevolutionary footprint, illustrating projections of the cumulative human macroevolutionary footprint on the mammal taxon by 2100 relative to a pre‐industrial reference, under the worst extinction scenario based on the IUCN Red List. Data are presented in Table 3. Representation of the three components of the macroevolutionary footprint: (1) variation in diversification dynamics, measured with extinction rate, speciation rate, and species richness; (2) changes in phylogenetic structure, measured using three groups of metrics following the framework of Tucker et al. (2017): richness, divergence, and regularity; and (3) the timescale of the driver's impact considered. The first temporality (T1) corresponds to realized impacts over anthropogenic time, typically decades to centuries, and in some cases millennia. The second temporality (T2) represents near‐future projections of macroevolutionary impacts of human drivers over eco‐evolutionary timescale, generally within the next 50 years. The third temporality (T3) encompasses long‐term evolutionary projections extending beyond 50 years. Values represent changes between the entity under the driver's pressure and in the reference scenario. Metric values are assigned to categories of magnitude of change, ranging from negligible (0) to very large (4). Diversification dynamics (D) and phylogenetic structure (S) are assigned the highest magnitude level among their internal metrics. The macroevolutionary footprint profile is expressed as the triplet {D, S, T}, with the associated magnitude level for each component (here: {D_Very large_, S_Medium_, T_3_}). The macroevolutionary footprint score is calculated as the mean of the scores of D and S. In this case, the D score is 4 and the S score is 2, resulting in a macroEvF score of 3.

Evaluating the magnitude of a driver's macroevolutionary footprint on a taxon implies focusing on three complementary levels: (i) changes in diversification dynamics, including variation in species richness and driver‐induced extinction and speciation rates; (ii) changes in phylogenetic structure, captured through phylogenetic diversity metrics within the focal taxon; and (iii) the timescale over which these changes have occurred (temporality). Together, these levels define a macroevolutionary footprint profile composed of changes in diversification dynamics (D), phylogenetic structure (S), and the timescale (T) over which these changes have occurred (Figure 3).

### 4.1 Measuring Changes in Diversification Dynamics

At macroevolutionary scales, drivers can modify species richness and extinction and speciation rates, thereby modifying diversification dynamics. These changes are expressed as deviations of extinction and/or speciation rates of a focal taxon from their background, pre‐driver rates, as inferred from paleontological data or diversification models. We quantify driver‐induced changes in macroevolutionary dynamics as the logarithmic ratio of extinction and speciation rates between the driver and reference scenarios. The absolute log‐ratio values are classified into five internal magnitude classes: negligible (< 0.7), small (0.7–1.6), medium (1.6–2.30), large (2.3–4.6), and very large (≥ 4.6) (Table 2). These thresholds, as done previously for the microevolutionary cases, are intended as pragmatic conventions rather than universal standards. They provide a quick view of relative change, notably distinguishing small deviations from background rate, substantial increases, severe crises, and mass‐extinction–level forcing. The diversification dynamics magnitude category (D) is defined as the highest magnitude class obtained across the extinction and speciation rate metrics (Table 2).

### 4.2 Measuring Changes in Phylogenetic Structure

Over deep time, extinctions and speciations have shaped biodiversity (Cowie et al. 2022) and generated the current tree of life and its associated phylogenetic diversity. Numerous phylogenetic metrics have been developed to characterize features such as phylogenetic diversity within and among taxa, the evolutionary distinctness of species, or other characteristics of phylogenetic trees. Tucker et al. (2017) proposed a comprehensive framework categorizing these metrics into three dimensions: richness, divergence, and regularity. Richness measures the sum of accumulated phylogenetic differences among taxa, divergence captures the mean phylogenetic relatedness among taxa, and regularity illustrates the variance in phylogenetic differences between taxa in an assemblage. The most common metrics used are Faith's phylogenetic diversity (PD) (Faith 1992) and evolutionary distinctiveness (ED) (Pavoine et al. 2005). The richness dimension is directly captured by PD and by the sum of species‐level ED values, which quantify the total amount of evolutionary history represented in a set of species. The divergence and regularity dimensions can then be derived from these basic metrics. Divergence in a taxon can be expressed, for example, as the mean phylogenetic diversity per species (avPD) or as the mean ED across species, whereas regularity can be summarized by the variance in species' ED values (var(ED)), reflecting how evenly evolutionary distinctiveness is distributed within the assemblage.

To assess driver‐induced changes in phylogenetic structure within the macroevolutionary footprint framework, we propose to quantify the percentage of change in the phylogenetic metrics (PM) of the focal taxon attributed to the driver's impact compared to a reference clade without driver‐induced extinctions and speciations. This quantification is performed for one PM in each of the three categories: richness, divergence, regularity (Table 2).

To use these PM values within a semi‐quantitative indicator, we classify them into five ordinal magnitude classes: negligible (0%–2%), small (2%–8%), medium (8%–20%), large (20%–40%), and very large (> 40%). Because there is no general consensus on what constitutes a “small” or “large” change in phylogenetic diversity, these magnitude classes we use are defined as pragmatic internal thresholds of the EvF scoring framework. They should be viewed as transparent conventions for comparative purposes rather than absolute or universal cut‐offs, and can be recalibrated as theory and additional empirical data accumulate.

We interpret changes in phylogenetic structure as the macroevolutionary analogue of changes in evolutionary potential at the microevolutionary scale, expressed in terms of evolutionary history and phylogenetic diversity. Beyond the direct loss or gain of inherited evolutionary history, deviations of phylogenetic diversity relative to a reference tree may also be interpreted as changes in “macroevolutionary potential”. The change in phylogenetic structure (S) is defined as the highest magnitude among the selected phylogenetic structure change metrics (Table 2).

### 4.3 Determining the Timescale of Macroevolutionary Changes

In addition to the magnitude of changes in macroevolutionary dynamics and patterns, the timescale over which divergence occurs is critical for interpreting the macroevolutionary footprint. For practical purposes, we distinguish three temporal scenarios commonly encountered in conservation context. The first temporality (T1) corresponds to realized impacts over past anthropogenic times, typically decades to centuries, and in some cases millennia. The second temporality (T2) represents near‐future projections of macroevolutionary impacts of human drivers over eco‐evolutionary timescale, generally within the next 50 years. The third temporality (T3) encompasses long‐term evolutionary projections extending beyond 50 years. To determine the macroevolutionary footprint expected in the future, we must consider the projection of future extinctions and their integration into the computation of phylogenetic metrics. This can be achieved, for example, by weighting species according to their extinction risk or threat status (Faith 2008; Veron et al. 2017).

### 4.4 Scoring the Macroevolutionary Footprint

To summarize, each driver–entity combination is characterized by a macroEvF scoring profile (D, S, T), composed of ordinal magnitude classes describing changes in diversification dynamics (D), changes in phylogenetic structure of the focal clade (S), and timescale category (T) over which these changes occurred. We further propose an integrative scoring method that condenses this triplet into a single numerical value ranging from 0 to 4, following an aggregation logic analogous to that used for the microevolutionary scale. Specifically, the diversification dynamics (D) and phylogenetic structure (S) scores—each ranked from 0 (negligible) to 4 (very large)—are averaged to represent the overall magnitude of the macroevolutionary impact. No weighting scheme is applied to the temporal component of the macroEvF, as the temporal categories (T1, T2, T3) reflect the observer's choice of analytical framework rather than intrinsic biological properties of the studied entity. However, the temporal category should be explicitly considered when interpreting the macroEvF triplet. A large evolutionary footprint associated with realized (T1) or near‐term projected (T2) impacts is of particular conservation concern, as it reflects past, ongoing or imminent macroevolutionary change that may be quickly prevented or mitigated. In contrast, long‐term projections (T3) are subject to greater uncertainty and allow more scope for adaptive management.

We illustrate this approach with a case study of the human‐induced macroevolutionary footprint in the mammal taxon (Box 2, Figure 3). In this example, the macroEvF profile is (D_Very Large_, S_Medium_, T_3_), corresponding to an overall macroEvF score of 3.

## 5 Discussion

### 5.1 Defining Thresholds for Evolutionary Metrics

Conceptualizing an evolutionary footprint (EvF) framework faces various challenges, inherent to the large diversity in biological entities and processes involved. The first issue we discuss here is the thresholds. Defining indicator thresholds is a longstanding challenge in ecology, particularly in conservation science (Huggett 2005; Johnson 2013; Spake et al. 2022). Thresholds are commonly used to delineate levels of impact on ecosystems and, in applied contexts, to justify limits on anthropogenic impacting activities. However, there is no consensus or standardized methodology for defining these thresholds. Some are intended to capture discontinuities or breakpoints in biological responses, whereas others aim to identify levels beyond which biological systems face a high probability of irreversible change, or a combination of both. Yet, recent research has raised concerns about the widespread use of thresholds in ecology, as ecosystems undergoing global change often lack the empirical data required to robustly identify such points (Hillebrand et al. 2020). It is therefore legitimate to question whether thresholds are necessary within the EvF framework. Nevertheless, despite these limitations, in conservation sciences—where decision‐making, prioritization, and categorization are central—the use of thresholds remains highly valuable, provided that they are transparent and open to revision as knowledge improves. Furthermore, the nature and consequences of evolutionary processes can vary greatly among species and contexts, making it particularly challenging to define universal thresholds for evolutionary footprint scores. This variability requires a case‐by‐case interpretation of EvF values and further underscores the importance of standardizing the measurement of evolutionary processes (Hansen et al. 2024).

### 5.2 Conceptualizing and Measuring Evolutionary Forcing

Evaluating the microevolutionary impact of a driver on a population or species requires assessing both the directionality of evolutionary change and its magnitude. To this end, the EvF framework proposed a definition for evolutionary forcing—a term that has rarely been used and lacks a consistent definition. Here, evolutionary forcing is defined as an integrative measure of the evolutionary pressure exerted by a driver on a focal entity. For practical purposes, the magnitude of evolutionary forcing is approximated by the highest observed quantitative change in trait mean or trait variance. However, strong evolutionary forcing does not necessarily correspond to statistically significant changes in trait distributions, nor does it need to be proportional to the severity of the driving factor itself. Instead, species‐specific ecological and evolutionary characteristics, as well as contextual factors, may generate distinct patterns such as evolutionary traps (Schlaepfer et al. 2002) or irreversible changes, which add an essential qualitative dimension to evolutionary forcing.

A key methodological choice of the EvF framework is to retain, for each trait category, the maximum observed magnitude of change in mean or variance, rather than averaging across traits. This deliberately maximalist approach is based on the assumption that a single strongly affected trait can be sufficient to substantially alter evolutionary trajectories. As a result, the EvF does not attempt to weight traits according to their relative contribution to fitness, nor to explicitly model trade‐offs or compensatory responses among traits. While this choice necessarily limits finer‐grained analyses of trait dynamics, it reduces the risk of diluting the impact of traits that are disproportionately important with respect to a given driver and greatly facilitates the scoring process for conservation practitioners. The microEvF score should therefore be interpreted as an indicator of maximum evolutionary pressure rather than as a detailed reconstruction of adaptive change, as illustrated by the *Viola arvensis* case study (Box 1).

### 5.3 Measuring Genetic Diversity and Evolutionary Potential

At the microevolutionary scale, quantifying and understanding genetic diversity change is crucial to implement an evolutionary footprint framework. Although a conceptual distinction is often made between adaptive and neutral diversity (Moritz 2002), recent studies suggest that, for conservation purposes, overall genomic diversity provides the most informative and robust indicator (Kardos et al. 2021). A reduction in genetic diversity thus represents a critical component of the EvF. Importantly, it also generates an EvF “debt”: beyond its immediate footprint, reduced diversity constrains future adaptation and increases the likelihood of subsequent evolutionary change or local extinction.

Methods for estimating intraspecific genetic diversity are numerous, as there is no single measure for population genetic diversity. According to Hughes et al. (2008), genetic diversity can be categorized into discrete allelic states and continuous traits. Key metrics for allelic state diversity include allelic diversity and richness, providing information about the frequency and number of alleles per locus; genotypic richness, indicating the number of haplotypes; heterozygosity, giving the average proportion of loci carrying two different alleles within an individual; nucleotide diversity, indicating the number of nucleotide differences per site between individuals; and percentage of polymorphic loci. Genomic advances in recent years have also made it possible to incorporate metrics such as heterozygosity‐rich regions as quantitative indicators of genetic variability (Biscarini et al. 2026).

A wide range of molecular markers can be used to measure neutral discrete allelic states and compute the genetic diversity component of the EvF. Although these markers may differ in terms of neutrality or non‐neutrality (Kirk and Freeland 2011) and definitions of intraspecific genetic diversity remain complex (Avolio et al. 2012), the EvF framework prioritizes the use of markers that maximize data quality and availability.

Despite the absence of a clear and consensual definition (Milot et al. 2020), we deliberately incorporated the concept of evolutionary potential into the EvF framework because of its central relevance for conservation. The use of evolutionary potential carries an inherent normative dimension, which requires explicit clarification. For practical reasons, evolutionary potential is represented here through a unidimensional metric of genetic diversity change. We acknowledge, however, that a comprehensive assessment of evolutionary potential should also integrate trait variation, ecological context, and the magnitude of phenotypic change, even if genetic diversity remains an efficient and operational indicator for conservation purposes (Hoban et al. 2024). Given these constraints, the unidimensional indicator proposed here cannot fully capture the complexity of evolutionary potential but provides a pragmatic approximation within the EvF framework.

### 5.4 Shortcomings in Categorizing Macroevolutionary Changes

Scoring the macroevolutionary footprint follows the same triplet logic as the microevolutionary footprint, but the two components cannot be interpreted symmetrically. At the microevolutionary scale, genetic diversity can provide a proxy for future adaptive potential, allowing the evolutionary potential score to carry a prospective, conservation‐relevant meaning. No such equivalent exists at the macroevolutionary scale: although losses of phylogenetic diversity can be quantified relatively straightforwardly, they cannot be straightforwardly interpreted as indicators of a future ‘macroevolutionary potential’. Predicting how current changes in phylogenetic structure will translate into future diversification dynamics remains highly uncertain, despite insights into the role of phylogenetic topology in shaping long‐term evolutionary trajectories (Box 2; Erwin 2008).

### 5.5 Scope of the EvF Framework

The scope of any EvF study is a capital issue, and in particular the choice of working scale (Schneider 2001), which encompasses temporal, spatial, and biodiversity dimensions. Evolution occurs continuously at all integration levels: from genes to ecosystems. However, evolutionary change is ultimately mediated through individual phenotypes and mostly transmitted across generations via genetic inheritance within populations. Consequently, micro‐EvF scoring is primarily conducted at the population level. Nevertheless, specific characteristics may warrant the operationality of the EvF at other levels of integration, infra‐population or species levels. At the macroevolutionary scale, evolutionary footprint can be assessed at levels where extinction, speciation, and their effects for phylogenetic structure can be observed, including sufficiently large taxa or biological assemblages such as biomes or isolated ecosystems. The concept of Evolutionarily Significant Units (ESUs) is particularly relevant in this regard. ESUs were originally defined as population units that are substantially reproductively isolated from other conspecific population units and therefore warrant distinct separate management or conservation consideration (Waples 1991). Over time, this concept has evolved to embrace a more integrative framework, incorporating phylogenetic, genomic, and ecological data (Hoelzel 2023). The EvF framework can build on these approaches to define focal entities, both below the species level and across higher taxa (Casacci et al. 2014), including contexts that extend beyond traditional conservation concerns.

Timing has been treated as a distinct dimension rather than being embedded directly within the metrics themselves. First, it allows the generation time of the focal species to be used as the basic temporal unit, ensuring alignment between timing categories and the biology of the taxon under study, including microorganisms for which microevolutionary change can be observed over very large numbers of generations. Second, categorizing timing into broad timescale classes is intended to correspond to the practical temporal horizons that are most relevant for common conservation scenarios and challenges.

### 5.6 Evolutionary and Non‐Evolutionary Responses to Driving Factors

Another, and arguably more challenging, issue concerns attributing observed biological changes to evolutionary processes and, in turn, attributing those evolutionary changes to the driver's impact. In many cases, such attribution is constrained by conceptual limitations or insufficient data, despite extensive theoretical and empirical work on genetic diversity, quantitative traits and species diversification (Bell 2008; Walsh and Lynch 2018). In principle, strong selective pressure will generate an EvF with varying magnitude. More particularly, a population with a high evolutionary potential may exhibit a strong evolutionary response in the presence of a strong driver, resulting in a pronounced microevolutionary footprint. Conversely, when evolutionary potential is low and evolutionary rescue is unlikely—defined as adaptive evolutionary change that restores positive population growth and prevents extinction (Carlson et al. 2014)—a strong driver may instead result in extinction, producing a macroevolutionary footprint. However, several caveats should be considered. Not all selective pressures lead to detectable evolutionary responses. This may reflect the nature of the traits under selection (e.g., correlation between traits), the detectability of the selection type (e.g., balancing selection), or methodological limitations in detecting evolutionary changes.

Detecting selective pressure requires a detailed understanding of interactions between the driver and the focal entity, correlations between traits of the focal entity as well as between traits and fitness (Hersch and Phillips 2004). This analysis cannot rely solely on traits already suspected to be under selective pressure; it also requires data on additional traits and environmental drivers. Detection power depends strongly on sample size. Hersch and Phillips (2004) suggest that a minimum sample size of several hundreds of individuals may be necessary to achieve adequate detection power. However, achieving such comprehensive coverage is rarely feasible. Broad coverage of various traits and evolutionary metrics is nonetheless essential, making it difficult to obtain the data required for precise EvF estimation, especially across long temporal spans involving numerous individuals and populations.

As a result, the EvF scoring framework is primarily suited to cases of directional selection, although changes in trait variance are also incorporated. Additionally, the absence of an observable evolutionary response does not imply the absence of evolutionary pressure. Evolutionary outcomes may be constrained, delayed, or masked by other biological processes. Moreover, observed phenotypic change under selection pressure does not always result from evolution, as they may instead result from other mechanisms such as phenotypic plasticity, defined as the ability of individual genotypes to produce different phenotypes when exposed to different environmental conditions (Pigliucci et al. 2006) and considered as a major driver of evolution (Sommer 2020), notably in the face of rapid environmental changes (Bonamour et al. 2019). Measuring an EvF therefore requires evidence that trait change has a genetic basis and that the focal driver is the causal selective force (Christie et al. 2023). Disentangling the contributions of multiple drivers is particularly challenging, given that the genetic basis of most traits remains poorly understood (Merilä and Hendry 2014). Consequently, there is a substantial “burden of proof” in evolutionary studies (Hansen et al. 2012). The debates surrounding changes in horn size in male bighorn sheep under selective trophy hunting pressure illustrate these challenges. Pigeon et al. (2016) argued that reduced horn size within the studied population resulted from artificial selection. Nevertheless, some authors considered that under human‐driven pressure, evolution plays a relatively minor role compared with phenotypic plasticity (Pelletier and Coltman 2018), and as a result, it might be disregarded in models developed to understand phenotypic changes (Coulson et al. 2017). Generally, the limiting factor is the lack of long‐term, high‐quality genetic data required to establish a firm causal relationship (Merilä and Hendry 2014). Some approaches allow stronger inference of genetic contribution of the observed change. In plants, for instance, the resurrection approach—illustrated by a field pansy case study in Box 1—compares ancestral and contemporary genotypes grown under common conditions to directly assess evolutionary change. Finally, the framework is also explicitly open to developments in genomics, which may offer more detailed and dynamic insights into the evolutionary processes than traditional marker‐based genetic approaches (Schmidt et al. 2024).

### 5.7 Aggregating Evolutionary Footprint Scores

Aggregation of evolutionary footprint (EvF) scores raises conceptual and methodological challenges. Aggregation can be performed across drivers or across focal entities and should be viewed as a synthetic representation of evolutionary pressure that supports comparison and prioritization rather than as an exact cumulative measure.

At the level of a given driver (e.g., land‐use practice or pollutant), aggregation would involve combining EvF scores across multiple affected entities. Because exhaustive coverage is rarely feasible, such aggregation would necessarily rely on representative sampling of taxa, species, or populations. A simple approach would be to calculate the arithmetic mean of the scores, although alternative weighting schemes based on exposure, abundance, or conservation relevance may be also appropriate, as commonly used in biodiversity indicators.

At the level of a focal entity, aggregation would combine EvF scores associated with multiple co‐occurring drivers. In this case, aggregation should account for the possibility that drivers may act independently or synergistically (positively or negatively). A simple and conservative approach would be to retain the maximum EvF score across drivers to identify the dominant evolutionary pressure. Alternatively, additive or weighted combinations could be used where empirical evidence supports cumulative effects of the different drivers. In conservation contexts, where the objective is often to represent the cumulative burden of impacts exerted by multiple drivers, summing scores can provide a synthetic measure of overall evolutionary pressure experienced by the entity, independently of whether the effects of different drivers are synergistic, antagonistic, or independent. In the case where evolutionary footprint is computed across multiple populations of a species, a key challenge is to infer the footprint at the metapopulation or species level. In such contexts, a similar weighting scheme could be applied, and the spatial scale, populations' distribution, and gene flows among populations should be assessed in order to upscale the population‐level footprint estimates.

Overall, aggregation within the EvF framework is intentionally flexible and purpose‐driven. Its implementation should depend on data availability, management objectives, and the scale of inference. Future work is needed to formalize aggregation rules, assess their sensitivity, and evaluate their relevance for conservation decision‐making.

### 5.8 Challenges in Data Collection and Availability

Computing reliable EvF scores requires substantial and specific data, highlighting the need to strengthen evolutionary and ecological knowledge relevant to biodiversity conservation. In particular, demonstrating that a given driver is the causal source of evolutionary pressure is a major challenge in microevolutionary studies and generally requires extensive data on trait variation, fitness, and their relationships with environmental drivers (Hersch and Phillips 2004). Such requirements are rarely fully met, especially across multiple traits, populations, and long temporal scales, which inevitably limits the precision of EvF assessments.

Data collection is further constrained by different biases. Research effort is unevenly distributed across taxa, with a strong bias towards large, emblematic, or human‐relevant species (Troudet et al. 2017) and across time (Bowler et al. 2025). Addressing these limitations will require research policy support, increased funding, data sharing among institutions and laboratories, and a systematic and coordinated global‐scale data collection efforts. Existing initiatives such as Essential Biodiversity Variables (Pereira et al. 2013), provide a valuable foundation for such efforts. EBVs are standardized biological state variables measured across time, space, and levels of organization, designed to monitor and understand biodiversity change, for example, species distribution, population abundance, age at maturity, and timing of life cycle events. Particularly relevant for the EvF framework is the proposed genetic extension to EBV (Hoban et al. 2022), which could facilitate the harmonization and aggregation of relevant evolutionary data.

Missing data is a crucial issue for any conservation frameworks. The EvF adopts a maximalist and precautionary approach, assuming that a single strongly affected component may be sufficient to capture the highest level of evolutionary impact exerted by a driver, and to jeopardize evolutionary trajectories or long‐term persistence. Such an approach is consistent with practices commonly adopted in conservation science, such as those of the IUCN Red List framework (IUCN 2023), where uncertainty and data deficiency often result in higher threat categories rather than underestimation of risk. However, in case of missing data or unevenly distributed across traits, genetic metrics, or taxa, the use of a maximum‐based aggregation can lead to an artificial inflation of footprint scores, as the absence of evidence for low‐impact dimensions does not counterbalance high values observed for others. Despite this drawback, we consider this conservative bias acceptable within the scope of the EvF, as underestimating evolutionary impacts would carry more severe consequences for conservation decision‐making than overestimating them. Nevertheless, future developments of the EvF framework should explicitly integrate confidence levels or data availability indices to better contextualize footprint values and guide their use in applied settings.

## 6 Perspectives

The framework proposed here primarily relies on commonly used phylogenetic and genetic‐centered metrics. Future developments will further integrate metrics related to cultural (Whiten 2019) and epigenetic (Ashe et al. 2021) evolutionary processes into the evolutionary footprint. Moreover, there is also a need for a more comprehensive understanding of the relationship between phenotypic plasticity and evolution (Merilä and Hendry 2014), along with insights into the reversibility of evolutionary processes, concepts such as evolutionary rescue, and the development and application of genomics (Heuertz et al. 2023). Evolutionary rescue occurs when adaptive evolution enables declining populations to recover growth rates, thereby avoiding their extinction (Carlson et al. 2014). This concept has undergone significant development as part of the field of eco‐evolutionary dynamics (Bell 2017). Incorporating evolutionary rescue into the EvF framework would allow a more nuanced projection of both micro‐ and macroevolutionary impacts of drivers, depending on species‐specific and ecological contexts. Experimental evidence could already help refine the concept of evolutionary debt.

One of the objectives of the EvF framework is to stimulate interest among both academics and conservation stakeholders—including government agencies, local and indigenous communities, non‐governmental organizations, and land managers, etc. through footprint scoring and the development of standardized references of type of driving factors. One subsequent step will then involve categorizing and quantifying driving factors, whether anthropogenic or not. Such an approach could enable the calibration of driver types, in order to identify and hierarchize the drivers with the most profound and lasting consequences for biodiversity. Although conservation science already relies on numerous groups of indicators, raising concern about redundancy, we argue that the EvF indicator could draw attention to evolutionary processes that are currently understudied or insufficiently considered. Ultimately, the EvF indicator is intended to be integrated into international frameworks for analysis and reporting in conservation sciences and policies.

To conclude, the EvF framework aims to promote an integrative and evolution‐centered approach in the life sciences. Recent conceptual and methodological advances have opened new opportunities for understanding evolutionary dynamics and interactions within living systems, particularly eco‐evolutionary interactions (Reznick et al. 2019; Bassar et al. 2021; Jarne and Pinay 2023; Yamamichi et al. 2023). This framework should be particularly useful for studying contemporary evolution in the Anthropocene, a period characterized by intensifying human impacts on biological processes and patterns (Palumbi 2001; Sullivan et al. 2017; Jørgensen et al. 2019). The evolutionary potential of species will play a critical role in determining species's responses to global changes. Urban et al. (2016) identified evolutionary potential as a key mechanism largely absent from current climate change predictive models, despite the development of eco‐evolutionary species distribution models integrating eco‐evolutionary dynamics (Lu et al. 2024; Thuiller et al. 2013). The EvF could therefore contribute to a framework in which evolution is explicitly incorporated as a mediator of responses to climate change (Urban et al. 2024). More broadly, it aims to promote an evolution‐informed approach to conservation and address a need among conservation practitioners (Cook et al. 2021).

The EvF framework serves various purposes and aligns with diverse scientific and ethical values. Understanding how species may evolve and adapt (or not) in response to human‐induced changes can be leveraged in the interest of humans to maintain ecological and evolutionary processes that benefit human societies (Faith et al. 2010; Forest et al. 2015) or to deliberately drive evolutionary change for human purposes (Carroll et al. 2014). Notably, the evolutionary consequences of gene editing and artificial selection driven by biotechnology (Kim et al. 2023) should be assessed and potentially regulated (Kofler et al. 2018; Li et al. 2021). From an ethical perspective, the EvF approach encourages the ongoing renewal of interactions involving humans and other‐than‐humans (IPBES 2022). Quantifying the magnitude and direction of human impacts on species' evolutionary trajectories can help reduce this footprint and foster evocentric conservation ethics (Sarrazin and Lecomte 2016; Genissel 2024; Genissel et al. 2025). Building on Soule's foundational principle for conservation (Frankel and Soulé 1981) while addressing contemporary challenges, this approach aims to alleviate the intense selection pressures exerted by humans on biodiversity and ultimately restore their evolutionary opportunities (Sarrazin and Lecomte 2016).

## Funding

This work benefited from the scientific context of the Tranloc Project funded through the 2020–2021 Biodiversa+ and Water JPI joint call for research projects, with the EU and the French ANR.

## Conflicts of Interest

The authors declare no conflicts of interest.

## Supporting information


**Supporting Information S1.** Categorization of driving factors (inspired by Battisti et al. 2016).

## Data Availability

Data sharing not applicable—no new data generated, or the article describes entirely theoretical research.
