# Supplementary material for: Evolutionary Footprint: A Systemic Indicator in Evolution, Ecology and Conservation
Source: Evol Appl. 2026 Jun 23;19(6):e70267. doi: 10.1111/eva.70267 (PMC13289801; doi:10.1111/eva.70267)
Supplement: Supplementary file 1 — Supporting Information S1. Categorization of driving factors (inspired by Battisti et al. 2016). [file EVA-19-e70267-s001.docx]

**Supplementary Material 1.** Categorization of driving factors (inspired by Battisti *et al.* *2016)*)

| Criteria | Attributes | Description | Example |
| --- | --- | --- | --- |
| Origin | *Biotic* | Mainly caused by living organism | Epidemic, Predation |
|  | *Abiotic* | Physical, chemical, or climatic event | Flood, fire, UV |
| Type | *Anthropic* | Human-induced factor | Light pollution |
|  | *Non-anthropic* | Non-human-induced | Beaver dams |
| Regime | *Discrete* | Distinct event in time | Meteorite impact |
|  | *Continuous* | Constant or almost constant pressure | Pollutant |
| Predictability | *Stochastic* | Unpredictable episodic or occasional factors | Drought, disease |
|  | *Deterministic* | Predictable periodic factors | Tides |
| Extension | *Local* | At site and landscape scale | Beaver dams |
|  | *Regional* | At continental and subcontinental scale | Volcanism |
|  | *Global* | At global scale | Climate change |
| Specificity | *Taxon* | Selective for a particular species or higher taxa | Pesticides |
|  | *Phenotype* | Selective for an intraspecific phenotype | Trophy hunting |
|  | *Non-specific* | Affects all or almost all entities | Temperature change |
| Severity | *Low* | Weak entity evolutionary response | Dependent on entity and driving factor |
